# Tumor-to-Tumor Metastasis in a Patient With Hemangioblastoma Unrelated to von Hippel-Lindau Disease: A Case Report and Review of the Literature

**DOI:** 10.7759/cureus.86881

**Published:** 2025-06-27

**Authors:** Kyoichi Tomoto, Kentaro Watanabe, Takahiro Ogawa, Hisao Sano, Hiroki Suetsugu, Ryoto Wachi, Michihiro Tanaka, Yuichi Murayama, Toshihide Tanaka

**Affiliations:** 1 Neurosurgery, Kameda Medical Center, Kamogawa, JPN; 2 Neurosurgery, The Jikei University School of Medicine, Tokyo, JPN; 3 Clinical Training Center, Tokyo Medical University, Tokyo, JPN; 4 Pathology, Kameda Medical Center, Kamogawa, JPN; 5 Neuroendovascular Surgery, Kameda Medical Center, Kamogawa, JPN

**Keywords:** breast cancer, collision tumor, hemangioblastoma, tumor-to-tumor metastasis, von hippel-lindau disease

## Abstract

Tumor-to-tumor metastasis (TTM) is a rare phenomenon in which one malignant tumor ("donor") metastasizes to another benign tumor ("recipient"). In the central nervous system (CNS), meningioma is considered one of the most frequent recipients, while hemangioblastoma (HGB) has also been reported, typically in association with von Hippel-Lindau (VHL) disease.

A 61-year-old woman with a history of bilateral breast cancer treated 17 years earlier presented with right-sided ataxic hemiparesis. Magnetic resonance imaging revealed a gadolinium-enhanced mass in the right cerebellum, initially diagnosed as a metastatic brain tumor. Despite stereotactic radiotherapy, symptoms persisted and the lesion enlarged. Surgical resection was performed via the occipital trans-tentorial approach. Intraoperative findings revealed a reddish, vascular tumor with a xanthochromic area. Complete resection was achieved. Pathological examination confirmed two distinct components: HGB and metastatic breast carcinoma, establishing the diagnosis of TTM. No evidence of VHL was identified. The pathogenesis of TTM remains unclear, although factors such as recipient tumor vascularity and a favorable microenvironment are proposed. The rarity of non-VHL-associated TTM in HGB emphasizes the need for further case accumulation, to clarify the underlying mechanisms.

We report the first description of TTM involving metastatic breast cancer to a sporadic HGB in the CNS, unrelated to VHL. This case highlights the importance of considering TTM among the differential diagnoses for CNS tumors, even in the absence of VHL.

## Introduction

A condition in which an individual shows two tumors at the same time is referred to as double cancer and is relatively rare. In some cases, one malignant tumor (the “donor”) metastasizes to another benign tumor (the “recipient”), in a phenomenon known as tumor-to-tumor metastasis (TTM; also called “collision tumor”). The first report of TTM in the central nervous system (CNS) was in 1930 [1], and approximately 150 cases have been documented since then. Meningioma is the most common primary tumor site, and breast cancer is one of the most frequent metastatic brain tumors causing TTM in the CNS [2,3]. The second most common recipient site is hemangioblastoma (HGB), with renal cell carcinoma, which was related to von Hippel-Lindau (VHL) disease, probably due to its vascularity with a favorable microenvironment to grow the different tumor clones in the same place. In contrast, TTM in non-VHL HGB is exceptionally rare, and mechanisms may differ from VHL-associated cases. Here, we present the first case of TTM in the CNS from breast cancer in a patient with sporadic HGB that was unrelated to VHL disease.

## Case presentation

Clinical course

A 61-year-old woman with a history of bilateral breast cancer treated 17 years earlier presented to our hospital with right-sided ataxic hemiparesis. The breast cancer had been positive for estrogen and progesterone receptors but showed low expression of human epidermal growth factor receptor 2. Following bilateral breast surgery, she had received additional radiotherapy and chemotherapy and was subsequently placed under regular follow-up. Although she had experienced a recurrence of breast cancer with supraclavicular lymph node and multiple bone metastases four years earlier, she had no other significant medical history. She also had no contributory family history.

**Figure 1:**
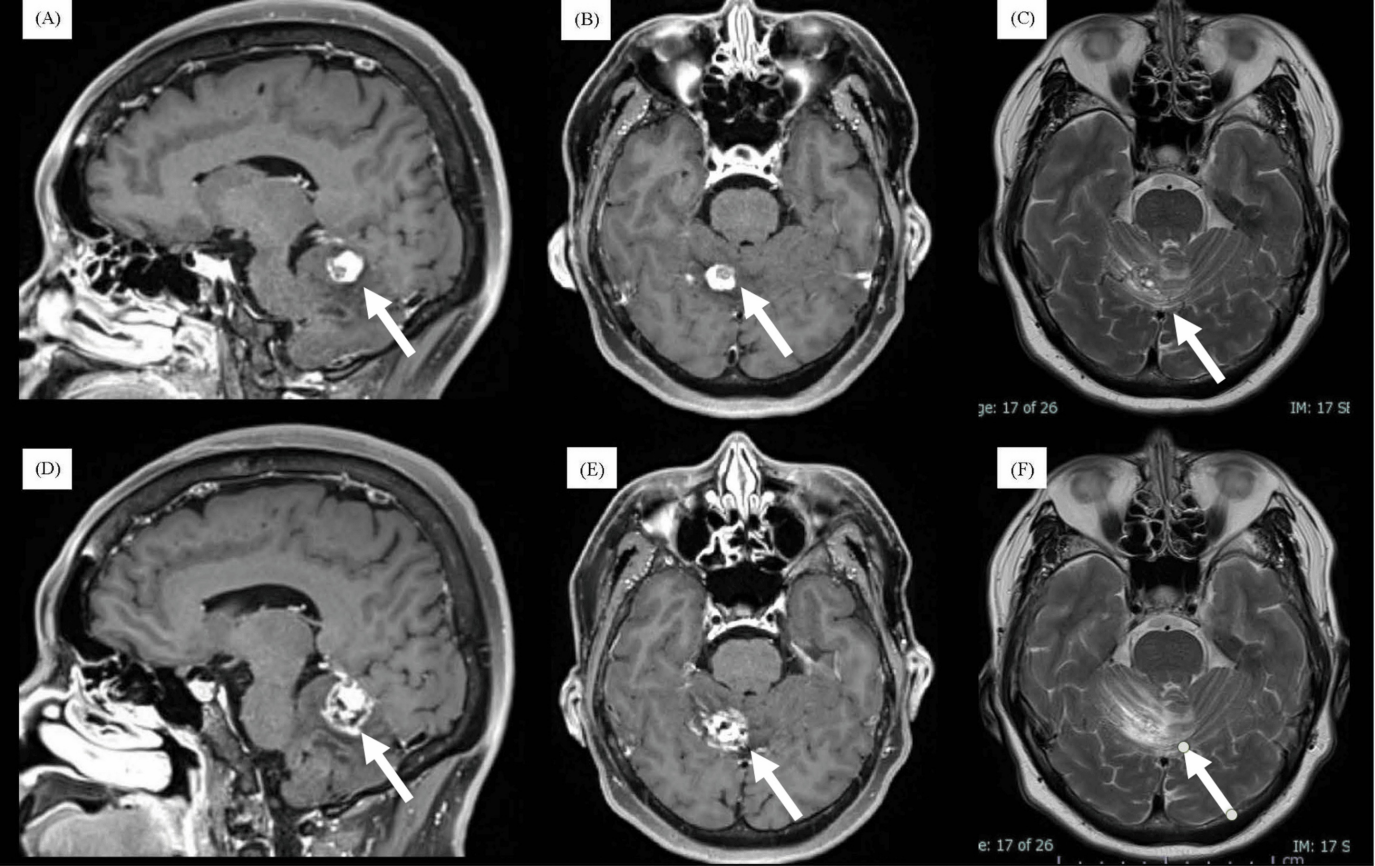
Pre-SRT (A-C) and post-SRT (D-F) MRI images (A-C) Pre-SRT MRI, showing a sagittal gadolinium-enhanced image (A), axial gadolinium-enhanced image (B), and axial T2-weighted image (C). A tumor with gadolinium enhancement is observed on the superior surface of the right cerebellum. (D-F) Post-SRT MRI (4 months later), showing a sagittal gadolinium-enhanced image (D), axial gadolinium-enhanced image (E), and axial T2-weighted image (F). SRT: stereotactic radiotherapy; MRI: magnetic resonance imaging

Magnetic resonance imaging (MRI) revealed a small, gadolinium-enhanced mass lesion on the tentorial side of the right cerebellum (Figure 1A). A non-enhancing area was observed in the anteromedial portion of the tumor, demonstrating perifocal edema. Oncologists initially diagnosed the lesion as a metastatic brain tumor and recommended stereotactic radiotherapy (SRT) without confirmation of histology. The patient underwent SRT (35 Gy in five fractions), but symptoms persisted and the tumor continued to grow along with increasing perifocal edema (Figure 1B). Characteristics of the non-enhancing area differed from those seen prior to SRT.

**Figure 2:**
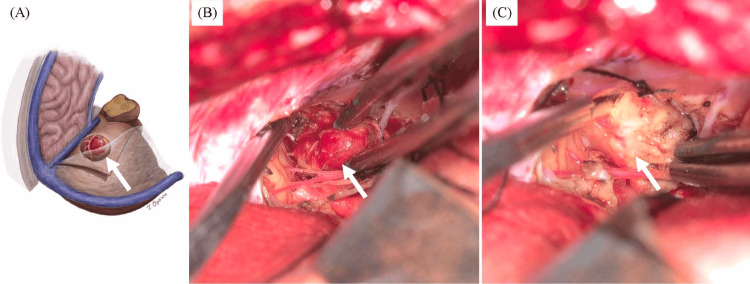
Illustration (A) and intraoperative photographs (B, C) during tumor resection via a right occipital trans-tentorial approach (A) Illustration of the reddish tumor and yellowish component before resection via the occipital trans-tentorial approach. (B) The reddish tumor emerging on the surface of the cerebellum during resection. The macroscopic appearance is suggestive of hemangioblastoma. (C) After removal of the reddish tumor, a yellowish component is resected. The boundary between the lesion and normal cerebellar tissue is clearly demarcated.

Given the lack of improvement and the growth of the non-enhancing area after SRT, we suspected the lesion represented HGB rather than a metastatic brain tumor from breast cancer. Eight months after the SRT, a cerebellar tumor containing reddish and yellowish components was removed via an occipital trans-tentorial approach (OTA) (Figure 2A). The reddish component formed a round and solid tumor with abundant blood supply (Figure 2B) and was resected en bloc. The surrounding yellowish component was clearly demarcated from the reddish component and was soft and poorly vascularized. The yellowish component was also distinctly different from adjacent normal cerebellar tissue (Figure 2C). Both components were completely removed.

Postoperatively, neurological symptoms improved completely, and the patient was discharged nine days after surgery. Postoperative imaging showed the total removal of the tumor.

Pathological findings

**Figure 3:**
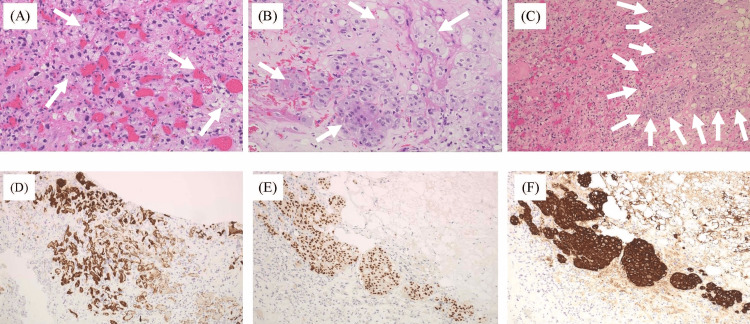
Histological findings of the surgical specimens (A) Hemangioblastoma component showing numerous foamy stromal cells and a rich capillary network (H&E staining, ×200). (B) Metastatic breast carcinoma component with hyperchromatic nuclei, eosinophilic cytoplasm, and some vacuolated cells (H&E staining, ×200). (C) Sharp boundary between hemangioblastoma (left) and metastatic breast carcinoma (right) (H&E staining, ×100). (D-F) Immunohistochemistry showing focal positivity for CD31 in hemangioblastoma (D) and for GATA3 (E) and AE1/AE3 (F) in metastatic breast carcinoma.

Histopathological examination confirmed the reddish mass as HGB and the peritumoral yellowish lesions as metastatic brain tumor with necrotic tissues originating from the previous breast cancer (Figure 3). The tumor turned out to have two components. First, most of the tumor tissue comprised HGB, revealing proliferation of cells with clear, foamy cytoplasm surrounding a well-developed capillary network (Figure 3A).

The second component of the tumor was identified as metastatic breast carcinoma (Figure 3B). These cells showed hyperchromatic, enlarged nuclei and eosinophilic cytoplasm, with some displaying vacuolation.

The boundary between these two components was clearly depicted (Figure 3C), with the left side corresponding to HGB and the right to metastatic breast cancer. Based on these findings, the tumor was diagnosed as a TTM with HGB.

Immunohistochemically, tumor cells with clear, foamy cytoplasm were focally positive for CD31 (Figure 3D), leading to the confirmation of the diagnosis. The Ki-67 labeling index was approximately 1%-2%, indicating low proliferative activity in the reddish component. We finally diagnosed the reddish compartment of the tumor as HGB in the CNS. Further investigations did not demonstrate a diagnosis of VHL disease. Tumor cells in the yellow component showed positivity for pancytokeratin AE1/AE3 (Figure 3E) and the common breast cancer markers GATA3 and GCDFP-15 (Figure 3F). Genetic sequencing was performed to definitively exclude VHL.

## Discussion

Here, we have presented the first description of TTM involving breast cancer metastasizing to HGB. Various reports have described TTM, but this pathology remains relatively rare in the CNS. So far, 29 metastases from other cancers to HGB in the CNS have been documented (Table 1) [4-25].

**Table 1:** List of metastatic brain tumors in the hemangioblastoma in the central nervous system RCC: renal cell carcinoma; VHL: von Hippel-Lindau

Number	Publication	Location	Primary tumor (donor)	Association with VHL
1	Crockard et al., 1988 [4]	Cerebellum	Prostate carcinoma	Not associated
2	Jamjoom et al., 1992 [5]	Cerebellum	RCC	Associated
3	Bret et al., 1999 [6]	Cerebellum	RCC	Associated
4	Mottolese et al., 2001 [7]	Cerebellum	RCC	Associated
5	Hamazaki et al., 2001 [8]	Cerebellum	RCC	Associated
6	Hamazaki et al., 2001 [8]	Spinal cord (thoracic)	RCC	Associated
7	Fakih et al., 2001 [9]	Spinal cord (cervical)	RCC	Associated
8	Abou-Hamden et al., 2003 [10]	Spinal cord (cervical)	RCC	Associated
9	Altinoz et al., 2005 [11]	Spinal cord (thoracic)	RCC	Associated
10	Jarrell et al., 2006 [12]	Cerebellum	RCC	Associated
11	Jarrell et al., 2006 [12]	Cerebellum	RCC	Associated
12	Jarrell et al., 2006 [12]	Spinal cord (thoracic)	RCC	Associated
13	Jarrell et al., 2006 [12]	Spinal cord (thoracic)	RCC	Associated
14	Jarrell et al., 2006 [12]	Spinal cord (sacrum)	RCC	Associated
15	Jarrell et al., 2006 [12]	Cerebellum	Pancreatic neuroendocrine tumor	Associated
16	Polydorides et al., 2007 [13]	Spinal cord (cervical)	RCC	Associated
17	Martin et al., 2010 [14]	Cerebellum	RCC	Associated
18	Xiong et al., 2010 [15]	Medulla oblongata	RCC	Associated
19	Ichikawa et al., 2011 [16]	Cerebellum	Mixed germ cell tumor	Not associated
20	Reynolds et al., 2015 [17]	Medulla oblongata, spinal cord (cervical)	Pancreatic neuroendocrine tumor	Associated
21	Dessauvagie et al., 2015 [18]	Cerebellum	RCC	Associated
22	Dessauvagie et al., 2015 [18]	Spinal cord (cervical)	RCC	Associated
23	Rai et al., 2015 [19]	Eye	RCC	Associated
24	Tran et al., 2021 [20]	Spinal cord (cervical)	RCC	Associated
25	Wakita et al., 2021 [21]	Spinal cord (thoracic)	RCC	Associated
26	Holanda and Lopes, 2022 [22]	Spinal cord (cervical)	RCC	Associated
27	Xie et al., 2023 [23]	Cerebellum	RCC	Associated
28	Lou et al., 2023 [24]	Ventricular triangle	RCC	Associated
29	Wang et al., 2024 [25]	Cerebellum	RCC	Associated
30	Present case	Cerebellum	Breast carcinoma	Not associated

Among these, HGBs were located in the cerebellum in 13 cases, the spinal cord in 13 cases, the medulla oblongata in two cases, and the eye and trigone of the lateral ventricular in one case each. The most common donor tumor is renal cell carcinoma, accounting for 25 cases. Other donors include pancreatic neuroendocrine tumor (two cases), as well as prostate carcinoma and mixed germ cell tumor (one case each). Of these 29 cases, only those involving prostate carcinoma or mixed germ cell tumor were unrelated to VHL disease. The remaining 27 cases were all associated with VHL disease. The present case represents the first to show TTM with HGB as the recipient tumor and breast cancer as the donor tumor.

The mechanisms underlying TTM have been discussed in various studies but remain unclear. Meningioma and HGB appear reasonable to consider as frequent donor sites due to their rich vascularity, but high vascularity alone does not fully explain this phenomenon. A well-nourished and slow-growing element is necessary in the recipient tumor to provide a favorable microenvironment for the donor tumor. This reasoning helps explain why gliomas are rarely reported as recipients despite their vascularity [26]. With respect to recipient tumors, meningiomas are often supplied by extra-cranial arteries, which disregard the protective effects of the blood-brain barrier and enable other tumors to metastasize. In contrast, HGBs are primarily supplied by blood flow from intracranial arteries, challenging this theory. Unlike meningiomas, which may be influenced by tumor vascularity, tumor vessels toward HGBs were derived from the intra-axial tumor.

In addition, for TTM involving meningiomas and breast cancer or VHL-related HGB, hormonal or genetic factors have been suggested as contributing mechanisms [25,27]. However, in the present case, we propose that an entirely different mechanism may be responsible. TTM should be considered in patients with a history of breast cancer and atypical CNS lesions, even without VHL.

Given the rarity of TTM involving non-VHL-related HGB, the accumulation of further cases is essential to clarify the underlying mechanisms. Understanding these mechanisms may not only provide insights into the pathophysiology of TTM but also help improve diagnostic and therapeutic strategies for such rare conditions.

## Conclusions

We encountered a rare case of TTM in which metastatic breast cancer metastasized to HGB in the CNS unrelated to VHL disease. This case expands the spectrum of donor tumors in TTM and underscores the importance of histopathological confirmation in atypical CNS lesions. TTM involving benign brain tumors such as meningioma is rare but should be considered among the differential diagnoses during clinical practice. Further accumulation of similar cases is needed to better understand the mechanisms underlying TTM to HGB not associated with VHL.
